# Depressive symptoms and social context modulate oxytocin’s effect on negative memory recall

**DOI:** 10.1093/scan/nsab072

**Published:** 2021-06-18

**Authors:** Shiu F Wong, Christopher Cardoso, Mark A Orlando, Christopher A Brown, Mark A Ellenbogen

**Affiliations:** Department of Psychology, Concordia University, Montreal, QC H4B 1R6, Canada; Department of Psychology, Concordia University, Montreal, QC H4B 1R6, Canada; Department of Psychology, Concordia University, Montreal, QC H4B 1R6, Canada; Adler University, Vancouver, BC V6B 3J5, Canada; Department of Psychology, Concordia University, Montreal, QC H4B 1R6, Canada

**Keywords:** intranasal oxytocin, autobiographical memory, depression, social context

## Abstract

Oxytocin promotes social affiliation across various species, in part by altering social cognition to facilitate approach behaviour. However, the effects of intranasal oxytocin on human social cognition are mixed, perhaps because its effects are context dependent and subject to inter-individual differences. Few studies have included explicit manipulations of social context to test this supposition. We examined oxytocin’s effects on autobiographical memory recall in two contexts, with and without social contact, and evaluated whether these effects were moderated by depressive symptoms. Two non-clinical samples (Study 1, *n* = 48; Study 2, *n* = 63) completed randomised, placebo-controlled, within-subject experiments. We assessed autobiographical memory recall across two sessions (intranasal oxytocin or placebo) and two contexts (memories elicited by an experimenter or by computer). Overall, intranasal oxytocin increased ratings of the vividness of recalled memories during the social context only. Individuals with elevated depressive symptoms also recalled memories that were more negative following oxytocin relative to placebo only in the non-social context across the two studies. Findings highlight the negative consequences of increasing oxytocin bioavailability in vulnerable persons in the absence of social contact. Contextual factors such as social isolation among depressed populations may complicate the clinical use of oxytocin.

Oxytocin is a mammalian hormone produced in the hypothalamus and is known for its role in a variety of social processes, including increased social affiliation and prosocial behaviour (Carter, 2014). The precise pathway through which oxytocin influences social behaviour is unclear, but researchers have proposed aspects of social cognition and decreased stress reactivity as possible mechanisms. Indeed, exogenous oxytocin administered using a nasal spray increases its levels in human cerebrospinal fluid (Striepens *et al.*, 2013) and activates brain circuits associated with social cognition and emotion processing within 25 min of administration (Paloyelis *et al.*, 2016). Intranasal oxytocin, relative to placebo, has been shown to increase trust and recognition of emotion in faces and to decrease stress reactivity in clinical populations (Kosfeld *et al.*, 2005; Ditzen *et al.*, 2009; Cardoso *et al.*, 2013, 2014a; Ellenbogen, 2018). Oxytocin also alters different aspects of self-perception. Studies have found that oxytocin reduces self-referential processing (biased processing of self-related *vs* other-related content) and promotes self-perceptions of extraversion, openness to new experiences, trust, altruism and communion (Cardoso *et al.*, 2012; Bartz *et al.*, 2015; Zhao *et al.*, 2016, 2020), all of which are associated with affiliative behaviour.

In addition, oxytocin might alter autobiographical memory recall. Individuals who self-administered intranasal oxytocin, relative to a placebo nasal spray, recalled less overgeneral memories (not based on a specific event or time point) and more specific personal memories and recalled social affiliation memories that were rated more positively (Cardoso *et al.*, 2014b). These oxytocin-induced changes in autobiographical memory recall might be highly relevant to studies of major depressive disorder (MDD), as persons with MDD report higher numbers of overgeneral memories than non-depressed persons (Raes *et al.*, 2006). Together, these findings suggest that intranasal oxytocin might be useful in targeting some of the core cognitive dysfunctions associated with MDD such as biased autobiographical memory recall.

However, many studies have reported weak or inconsistent results when examining the main effect of oxytocin on social behaviour and cognition (Bartz *et al.*, 2011; Tabak *et al.*, 2019). A recent well-powered replication of the seminal study on trust by Kosfeld *et al.*, (2005), for example, failed to detect a significant change in trust following oxytocin administration (Declerck *et al.*, 2020). In a review by Bartz *et al.*, (2011), three out of six intranasal administration studies reported non-significant effects of oxytocin on emotion recognition or empathy, and almost 50% of studies found no significant main effects of oxytocin on prosocial outcomes (e.g. generosity and cooperation). Some studies also reported increased feelings of envy (Shamay-Tsoory *et al.*, 2009), mistrust (Declerck *et al.*, 2010; Bartz *et al.*, 2010a) or attachment insecurity (Bartz *et al.*, 2010c) following intranasal oxytocin administration, relative to placebo. These conflicting results have also been observed in studies of memory, with some reporting that oxytocin facilitated recall of positive stimuli (Guastella *et al.*, 2008; Di Simplicio *et al.*, 2009) and others the recall of negative stimuli (Striepens *et al.*, 2012; Weigand *et al.*, 2013).

Given these inconsistencies, the relationship between oxytocin and social outcomes may be constrained by contextual and/or individual difference factors (Bartz *et al.*, 2011; Brown *et al.*, 2016). In most of the studies reviewed by Bartz *et al.*, (2011), they found evidence that the relationship between oxytocin, social cognition and prosociality was moderated by factors such as task difficulty (Domes *et al.*, 2007), social competence (Bartz *et al.*, 2010b), the presence of a psychological disorder (e.g. borderline personality disorder) or an anxious attachment style (Bartz *et al.*, 2010a). Although they reported no main effects of oxytocin on trust, Declerck *et al.*, (2020) found that oxytocin increased trust for individuals with a low disposition toward trust. Similarly, male participants low on social competence improved empathic accuracy (concordance between participants’ ratings of emotion with those of the person experiencing the emotion) following oxytocin administration relative to placebo (Bartz *et al.*, 2019). Other studies have found that deficits in social functioning might represent a vulnerability for possible negative effects following oxytocin administration, as observed in persons who are insecure, self-critical, lonely, sad or less prone to engaging with others (Bartz *et al.*, 2011; Norman *et al.*, 2011; Rockliff *et al.*, 2011; Ellenbogen *et al.*, 2013; Wong *et al.*, 2021). Thus, inter-individual differences might be critical in determining who might benefit from intranasal oxytocin administration.

Elevated depressive symptoms might represent an important individual difference factor that influences the response to intranasal oxytocin challenges. Elevated depressive symptoms, which indicates risk for developing MDD (Klein *et al.*, 2013), moderated the relationship between oxytocin administration and early (automatic) processing of masked angry faces on an attentional shifting task (Ellenbogen *et al.*, 2012). That is, oxytocin facilitated disengagement from masked angry faces (i.e. reduced attentional bias) in dysphoric participants but increased attentional bias in individuals with low depressive symptoms. In another study, participants with elevated depressive symptoms demonstrated a sharp decline in their ability to ignore sad faces on a negative affective priming task when administered oxytocin relative to placebo (Ellenbogen *et al.*, 2013). Both studies demonstrated an increased sensitivity to exogenous oxytocin in persons reporting depressive symptoms.

Contextual factors might also be important in understanding how oxytocin influences social behaviour. For example, exogenous oxytocin administration can promote negative behaviours such as outgroup aggression in competitive or conflictual contexts (De Dreu *et al.*, 2011, 2012), perhaps as a deleterious consequence of increased in-group solidarity. Context-dependent effects might also be understood by the hypothesis that oxytocin increases the social salience of social cues regardless of the valence of those cues (Shamay-Tsoory and Abu-Akel, 2016). For example, intranasal oxytocin, relative to placebo, elicited increased self-reported hostility in male and female participants who were provoked by a rude and ambivalent confederate during participation in a behavioural aggression task (Romney *et al.*, 2019).

A recent study demonstrated that contextual and inter-individual factors can interact with oxytocin in affecting social affiliation (Cardoso *et al.*, 2016b). Using a within-subjects and counterbalanced design, participants attended two test sessions where they were randomly assigned to self-administer oxytocin or placebo, before being asked to recall emotional autobiographical memories with and without social contact with a same-sex experimenter. When social contact with the experimenter was absent, participants who self-administered oxytocin, relative to placebo, had lower ratings of perceived experimenter support (on an in-house measure) during recall of negative memories. In this non-social context, women who were also motivated to affiliate with the experimenter following oxytocin administration showed this effect in greater magnitude, whereas this effect was reversed (i.e. increased perceived support in response to oxytocin) when social contact with the experimenter was available. Male participants did not demonstrate this pattern. These results, and those of others (Romney *et al.*, 2019), suggest that increasing the bioavailability of oxytocin in the absence of social support could be detrimental and that a positive social outlet may be necessary to elicit the prosocial effects of oxytocin.

## Aims and hypotheses

The present studies aimed to assess the effects of intranasal oxytocin, relative to placebo, on autobiographical memory recall during a standard cued recall of autobiographical memories administered under social and non-social conditions. In this study, we assessed three aspects of episodic memory recall: valence, vividness and the extent of recalling overgeneral memories. Memory vividness was of interest since previous research has shown that this may increase as negative emotion increases (Cooper *et al.*, 2019) and that it might be influenced by oxytocin administration. Hu *et al.*, (2019) found that oxytocin administration in humans, relative to placebo, facilitated the extinction of a conditioned threat memory a day after this memory was retrieved without the presentation of the threat (i.e. extinction learning). As oxytocin administration did not block the reconsolidation of the threat memory following retrieval (Hu *et al.*, 2019), it is possible that this extinction-enhancing effect was due to oxytocin increasing the vividness of the extinction learning memory.

Given the importance of inter-individual factors and context in understanding oxytocin’s effects on social behaviour and cognition, we assessed whether the interaction between intranasal oxytocin administration and participants’ depressive symptoms on autobiographical memory recall varied across a social and non-social context. This research question was investigated using data from two studies. Study 1 examined the relationship between depressive symptoms and autobiographical memory recall following oxytocin administration, relative to placebo, when the memories were elicited by computer administration, devoid of social contact. Study 2 similarly examined the relationship between depressive symptoms and autobiographical memory recall following oxytocin or placebo administration, except that memory recall was assessed in two contexts, alone by computer or with an attentive research assistant.

Two hypotheses were put forth. First, we predicted that oxytocin, relative to placebo, would elicit increased negative autobiographical recall (increased negative valence, decreased vividness and more overgeneral memories) in the non-social context compared to the social context. That is, we predicted that Study 1 would show a negative oxytocin effect, and Study 2 would show a drug by context interaction, where oxytocin would show more positive effects in the social condition compared to the non-social condition. Second, we hypothesised that persons with elevated depressive symptoms will exhibit greater negative autobiographical memory recall following oxytocin, relative to placebo, in the negative social context than persons with few or no depressive symptoms.

In the interest of transparency of scientific reporting, Study 1 is based on data collected in a sample reported by Cardoso *et al.*, (2016a), where we reported on the frequency of social affiliation and social conflict memories as predictors of relationship quality over an 18 month period. Study 2 utilises data from Cardoso *et al.*, (2016b), where we reported on ratings of emotional support during autobiographical memory recall and ratings of likeability towards the experimenter during the experimental procedures. Importantly, the data reported in the present manuscript are novel and have not been published in these previous publications.

## Study 1

### Method

#### Participants.

Forty-eight participants (24 female) between the ages of 19 and 32 years (*M* = 23.71, s.d. = 3.52) were recruited through online advertisements (e.g. Craigslist). Exclusion criteria included current use of tobacco or prescription drugs, current or past use of illicit drugs (excluding cannabis, which required 1-year abstinence), severe medical conditions, history of receiving psychiatric or psychological services and suspected pregnancy in women. Only participants who were fluent in English were included in the study. To achieve a greater range of depression in this healthy non-clinical sample, oversampling on the Beck Depression Inventory—Second Edition (Beck *et al.*, 1996) was employed, with 25% of participants reporting mild-to-severe depressive symptoms (scores ≥ 14).

#### Materials and measures

##### Beck depression inventory—second edition (Beck et al., 1996).

The Beck depression inventory—second edition (BDI-II) is a 21-item self-report questionnaire measuring the severity of depressive symptoms experienced during the previous 2 weeks. It has excellent internal consistency (Cronbach’s α = 0.92–0.93; Beck *et al.*, 1996) that was comparable to the current sample (Cronbach’s α = 0.93). BDI-II scores ranged from 0 to 30 (*M* = 9.58, s.d. = 9.23).

##### Autobiographical memory test (Cardoso et al., 2014b).

The autobiographical memory test (AMT) is based on the well-established procedures reported by Williams and Broadbent (1986). Participants were asked to recall specific episodic memories (event contained within a single 24-hour period, e.g. ‘When I was camping last weekend, my friend cooked me breakfast Saturday morning’) in response to positive-, negative- and neutral-valenced cue words. Cue words were presented visually on a computer to participants, who were alone in a room that was being monitored remotely. Examples of positive cue words included ‘happy’, ‘proud’ and ‘brave’; negative cue words included ‘lonely’, ‘angry’ and ‘afraid’ and neutral cue words included ‘radio’, ‘river’ and ‘chair’. Participants recalled memories in response to 78 unique cue words in each test session that alternated between positive, negative and neutral valence. As such, 156 cue words were randomised into two word lists that were counterbalanced across two test sessions (oxytocin and placebo). After recalling each memory, participants rated (i) how positive the memory was; (ii) how negative the memory was and (iii) the vividness of the memory on 0- to 100-point visual analogue scales (0 = not at all and 100 = extremely. Participants also rated (iv) how often they think of this memory on a 0- to 100-point visual analogue scale (0 = not at all and 100 = all the time). Finally, as participants also often recall overgeneral memories in response to these cues (event spanned more than 24 hours, e.g. ‘I went camping with my friends last weekend’), two independent raters coded memories as either specific or overgeneral. As we had no specific hypotheses on the positive valence of memories and memory frequency, these data are not reported.

#### Procedure.

Eligible participants were scheduled for two laboratory visits 1 week apart, with the time of day consistent across test sessions. Female participants were scheduled during the luteal phase of their menstrual cycle as to control for natural hormonal variations. Accurate scheduling within this phase was achieved by having females contact the laboratory on the first day of their menstruation.

Prior to their first test session, participants completed the BDI-II. Upon arrival, participants were first prepared for electroencephalography (EEG) data acquisition, which is not reported here. Briefly, this involved positioning the EEG cap according to standardised procedures (Pivik *et al.*, 1993) and skin preparations (i.e. gently abrading the scalp at electrode sites and adding electrolyte gel). Participants then self-administered 24IU intranasal oxytocin (*Syntocinon*, Novartis) or a placebo with matched inactive ingredients, with the order of drug administration across both test sessions counter-balanced across participants. Drug administration was conducted in accordance with published guidelines on intranasal oxytocin administration (Guastella *et al.*, 2013). This was followed by having participants rest in a chair and reading neutral magazines for 30 min. Participants then underwent a baseline EEG recording for 8 min and, then, completed the AMT alone in the room administered remotely by a computer during EEG acquisition. At the end of the first session, participants were scheduled for the second test session, which followed the same procedure (but self-administration of the opposite compound). At the end of the second test session, participants were debriefed and remunerated $80 CAD. This project was approved by the Human Research Ethics Committee at Concordia University (Montreal, Canada). Informed written consent was obtained for all participants.

### Results and discussion

#### Drug and sex effects on memory characteristics.

Using IBM SPSS v.27, we conducted a 2 (drug: oxytocin and placebo) × 2 (sex: male and female) × 3 (memory cue: positive, negative and neutral) repeated measures multivariate analysis of variance (MANOVA), with drug and memory cue as the within-subject independent variables, sex as the between-subject independent variable and the memory characteristics assessed on the AMT as the dependent variables (negative valence of the memory, memory vividness and the number of overgeneral and specific memories). As cue type did not significantly interact with drug, *F*(8, 38) = 0.79, *P* = 0.62, *η^2^_p_* = 0.14 or the drug × sex interaction, *F*(8, 38) = 1.12, *P* = 0.38, *η^2^_p_* = 0.19, this was dropped from the model to increase statistical power and for simplicity of interpretation.

Contrary to prediction, there was no significant overall main effect of drug, *F*(4, 42) = 0.20, *P* = 0.94 and *η^2^_p_* = 0.02. Results of separate univariate analyses of variance (ANOVAs) of the drug main effect across memory characteristics are presented in Table 1. There was no significant overall main effect of sex, *F*(4, 42) = 1.82, *P* = 0.14, *η^2^_p_* = 0.15 and no significant overall drug × sex interaction effect *F*(4, 42) = 0.82, *P* = 0.52, *η^2^_p_* = 0.07.

**Table 1.** Comparing drug effect on memory characteristics in non-social and social contexts (Studies 1 and 2)

		Drug						
		Oxytocin		Placebo				
		*M*	s.d.	*M*	s.d.	*F*(45)	*P*	*η^2^_p_*
Study 1 (*n* = 48)								
Non-social context	Negative valence	36.41	9.04	35.87	9.25	0.24	0.63	0.01
	Vividness	64.41	14.71	62.94	15.18	0.72	0.40	0.02
	# Overgeneral	13.32	8.30	13.47	7.32	0.02	0.89	<0.001
	# Specific	57.15	13.05	57.30	12.67	0.01	0.91	<0.001
Study 2 (*n* = 63)						*F*(61)		
Non-social context	Negative valence	2.70	0.61	2.70	0.67	0.003	0.96	<0.001
	Vividness	4.89	1.05	4.88	1.04	0.02	0.88	<0.001
	# Overgeneral	6.56	4.03	6.24	3.85	0.38	0.54	0.01
	# Specific	22.17	5.46	22.30	5.37	0.04	0.84	0.001
Social context	Negative valence	2.59	0.63	2.60	0.58	0.03	0.86	0.001
	Vividness	**5.03**	**1.03**	**4.90**	**1.10**	**5.30**	**0.03**	**0.08**
	# Overgeneral	6.41	3.70	6.57	3.76	0.11	0.74	0.002
	# Specific	22.08	5.07	22.35	4.85	0.27	0.60	0.004

Bold values indicate significance.

# means number of.

#### BDI-II scores moderating the drug effect on memory characteristics.

Four linear regression models were conducted using IBM SPSS v.27 to examine whether self-reported depression on the BDI-II moderated the relationship between drug administration and memory characteristics on the AMT. That is, the drug effect on each memory characteristic was calculated to a difference score (oxytocin minus placebo), and these were predicted by the BDI-II in separate models (Table 2). Sex was not included in these models because of the lack of a significant drug × sex interaction effect in the MANOVA reported above. As expected, higher levels of depressive symptoms significantly predicted a positive drug effect (oxytocin > placebo) on the negative valence ratings of memories (effect size: *R^2^* = 0.11). That is, persons with higher depression scores reported memories that were more negatively valenced following intranasal oxytocin administration compared to placebo during a computer administration of the autobiographical memory task.

**Table 2.** Drug effect on memory characteristics moderated by depression scores (Studies 1 and 2)

		Predictor	*R^2^*	*B*	*SE B*	β	*P*
Study 1 (*n* = 48)							
Non-social context	Drug effect on memories negative valence	BDI-II	**0.11**	**0.29**	**0.12**	**0.34**	**0.02**
	Drug effect on memory vividness	BDI-II	0.05	0.28	0.18	0.23	0.13
	Drug effect on # of overgeneral memories	BDI-II	0.01	0.08	0.10	0.12	0.42
	Drug effect on # of specific memories	BDI-II	0.03	0.15	0.13	0.17	0.25
Study 2 (*n* = 63)							
Non-social context	Drug effect on memories negative valence	BDI-II	**0.05**	**0.02**	**0.01**	**0.23**	**0.07**
	Drug effect on memory vividness	BDI-II	0.01	0.01	0.01	0.09	0.51
	Drug effect on # of overgeneral memories	BDI-II	<0.001	−0.01	0.09	−0.02	0.89
	Drug effect on # of specific memories	BDI-II	0.02	0.12	0.11	0.13	0.30
Social context	Drug effect on memories negative valence	BDI-II	0.01	−0.01	0.01	−0.08	0.56
	Drug effect on memory vividness	BDI-II	0.03	0.01	0.01	0.18	0.16
	Drug effect on # of overgeneral memories	BDI-II	0.001	−0.02	0.08	−0.03	0.84
	Drug effect on # of specific memories	BDI-II	0.003	0.04	0.08	0.06	0.66

*Notes:* Drug effect for each memory characteristic was calculated by subtracting placebo from oxytocin.

These findings support the hypothesis that oxytocin has negative effects when administered in an aversive context (De Dreu *et al.*, 2011, 2012; Cardoso *et al.*, 2016b), perhaps as a function of increasing social salience when there are no appropriate social outlets or only negative social cues are available. These results are consistent with previous research showing that participants with depressive symptoms were unable to inhibit the processing of sad faces during a cognitive inhibition task following oxytocin administration compared to placebo (Ellenbogen *et al.*, 2013), which is similar to oxytocin’s potentiation of negatively valenced autobiographical memories in the present study. Although we speculate that the autobiographical memory task administered by computer during an EEG protocol inadvertently created an aversive context, considering the absence of social contact and the discomfort associated with wearing a tightly fitted electrode cap for a few hours, Study 1 did not manipulate context. Thus, Study 2 addressed this limitation by explicitly manipulating context. In this randomised double-blinded within-subjects crossover study, participants were administered intranasal oxytocin or placebo and then completed two versions of the autobiographical memory task in random order: administered by an attentive research assistant or administered via computer. As in Study 1, we report on the characteristics of autobiographical memories collected through the autobiographical memory task.

## Study 2

### Method

#### Participants.

Sixty-three participants (32 female) between the ages of 18 and 35 years (*M* = 24.60, s.d. = 4.22) were recruited through online advertisements (e.g. Craigslist). Exclusion criteria included current use of tobacco or prescription drugs, current or past use of illicit drugs (excluding cannabis, which required 1-year abstinence), severe medical conditions, history of receiving psychiatric or psychological services and suspected pregnancy in women. Only participants who were fluent in English were included in the study. Of the 25 females not taking the oral contraceptive pill, six were in the follicular menstrual phase and 19 were in the luteal phase across all test sessions.

#### Materials and measures.

Internal consistency of the BDI-II in the current sample was good (Cronbach’s α = 0.85), with scores ranging from 0 to 35 (*M* = 7.52, s.d. = 6.21). We used a modified version of the AMT that manipulated social context (Cardoso *et al.*, 2016b) by having cue words verbally presented to participants by a same-sexed experimenter in-person (social condition) or visually presented on a computer to participants who were alone in a room that was being monitored remotely (non-social condition). Participants recalled memories in response to 30 unique cue words in each condition that alternated between positive, negative and neutral valence. As such, 120 cue words were randomised into four word lists that were counterbalanced across both conditions (social and non-social) and across two test sessions (oxytocin and placebo). Similar to Study 1, two independent raters coded memories as either specific or overgeneral and participants in the current study rated the positive and negative valence of each recalled memory, as well as the vividness and frequency of each memory (kappa = 0.97). Unlike Study 1, however, these ratings were made on 1- to 7-point scales (1 = not at all and 7 = extremely/all the time). As in Study 1, memory frequency and positive valence of the memories are not reported.

#### Procedure.

Eligible participants were scheduled for two laboratory visits 1 week apart, with the time of day consistent across test sessions. Females were scheduled for both sessions on days that they were taking the active oral contraceptive pill to control for variations in oestrous hormones. Females not taking the oral contraceptive pill were scheduled for both testing sessions within 0–11 or 17–25 days of the first day of menstruation. Accurate scheduling within either phase was achieved by having females contact the laboratory on the first day of their menstruation.

Prior to their first test session, participants completed the BDI-II. Upon arrival, participants self-administered 24IU of intranasal oxytocin or placebo, which was followed by a 30 min rest period (see Study 1 procedure). The order of drug administration was counter-balanced across participants. Participants then completed a 60 min eye-tracking experiment, which is not reported here, before being administered the social and non-social AMT described above. The order of test conditions on the AMT (social, non-social) was counter-balanced across participants. At the end of the first session, participants were scheduled for the second test session, in which they self-administered the opposite drug and then completed the social and non-social AMT. At the end of the second test session, participants were debriefed and remunerated $70 CAD. This project was approved by the Human Research Ethics Committee at Concordia University (Montreal, Canada). Informed written consent was obtained for all participants.

### Results and discussion

#### Drug and sex effects on memory characteristics.

Within each context, using IBM SPSS v.27, we conducted a 2 (drug: oxytocin and placebo) × 2 (sex: male and female) × 3 (memory cue: positive, negative and neutral) repeated measures MANOVA, with drug and memory cue as the within-subject independent variables, sex as the between-subject independent variable and the memory characteristics assessed on the AMT as the dependent variables (negative valence of the memory, memory vividness and the number of overgeneral and specific memories). As observed in Study 1, cue type did not significantly interact with drug or the drug × sex interaction in either context (*P*’s > 0.05) and was dropped from the models to increase statistical power and for simplicity of interpretation.

In the non-social condition, there was no significant overall main effect of drug, *F*(4, 58) = 0.17, *P* = 0.95, *η^2^_p_* = 0.01, sex, *F*(4, 58) = 0.92, *P* = 0.46, *η^2^_p_* = 0.06 or drug × sex interaction, *F*(4, 58) = 0.99, *P* = 0.42, *η^2^_p_* = 0.06. In the social condition, there was no significant overall main effect of drug, *F*(4, 58) = 2.34, *P* = 0.07, *η^2^_p_* = 0.14 or drug × sex interaction, *F*(4, 58) = 1.12, *P* = 0.36, *η^2^_p_* = 0.07. However, the overall main effect of sex was significant, *F*(4, 58) = 2.55, *P* = 0.05, *η^2^_p_* = 0.15. Separate univariate ANOVAs of the sex main effect across memory characteristics in the social condition revealed that averaging across drug, females reported significantly more vivid memories (*M* = 5.23, s.d. = 1.09) than males (*M* = 4.70, s.d.* = *0.97), *F*(1, 61) = 4.41, *P* = 0.04, *η^2^_p_* = 0.07. Results of separate univariate ANOVAs of the drug main effect across memory characteristics revealed that the intranasal administration of oxytocin in the social condition, relative to placebo, significantly increased ratings of vividness of autobiographical memories recalled, *F*(1, 61) = 5.30, *P* = 0.03, *η^2^_p_* = 0.08 (Table 1).

#### BDI-II scores moderating the drug effect on memory characteristics.

**Fig. 1.**
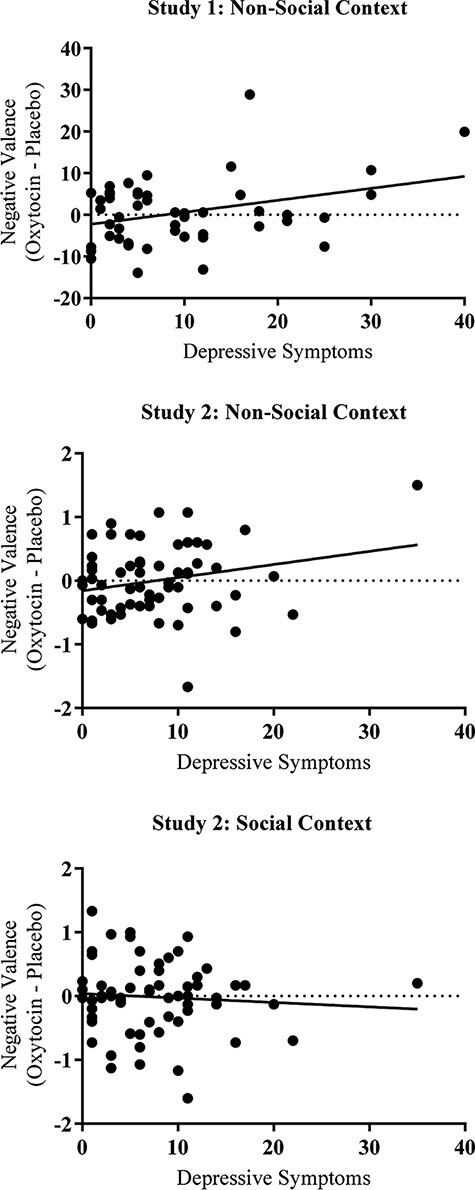
Drug effect on the negative valence of memories in a non-social *vs* social context.

Within each context, hierarchical multiple regression models were conducted using IBM SPSS v.27 to examine whether self-reported depression on the BDI-II moderated the relationship between drug administration and memory characteristics on the AMT. That is, the drug effect on each memory characteristic in each condition was calculated into a difference score (oxytocin minus placebo) and these were predicted by the BDI-II in separate models (Table 2). Sex was not included in these models because of the lack of significant drug × sex interaction effects in the MANOVAs reported above. Similar to Study 1, higher levels of depressive symptoms predicted a positive drug effect (oxytocin > placebo) on the negative valence ratings of memories in the non-social condition (effect size: *R^2^* = 0.05), with the effect falling just short of the conventional level of statistical significance. In contrast, depressive symptoms had no effect on negative valence ratings in the social condition (effect size: *R^2^* = 0.01; Figure 1).

Two key findings emerged. First, the administration of intranasal oxytocin elicited memories that were rated as being more vivid relative to memories elicited in the placebo condition but only in the social condition where autobiographical memories were elicited by an attentive research assistant. Like Study 1, oxytocin had no effect on ratings of vividness during memory recall in the non-social condition, when the task was administered via a computer. These findings parallel the work in non-human animals showing that oxytocin heightens the emotional salience of social memories (e.g. Guzmán *et al.*, 2013, 2014). Second, across two studies, persons with high depression scores reported more negatively valenced memories following intranasal oxytocin administration compared to placebo when the autobiographical memory task was administered alone by computer, with no social contact. In contrast, the effect was absent when a research assistant administered the task. Although the examination of effect sizes supports this interpretation, the drug by depressive symptoms interaction fell short of the conventional level of statistical significance, unlike in Study 1.

One reason for the weaker effect in Study 2 may be due to methodological differences between studies. For instance, participants in Study 1 underwent an EEG protocol during the autobiographical memory test while those in Study 2 did not. The Study 1 protocol was lengthy (around 2 h) and likely elicited physical discomfort from the tight-fitting EEG cap, thus creating a more aversive non-social environment than the one in Study 2. As this is speculation, future replications of the current research should have participants rate perceived displeasure and discomfort during the experiment.

Another difference between studies was the timing of the AMT following oxytocin administration. In Study 1, the AMT began after a 30 min rest period and an 8 min EEG calibration following administration, whereas in Study 2, this rest period and a 60 min eye-tracking experiment (i.e. 90 min) was between administration and the AMT. This later timing of the AMT along the drug time course may have resulted in weaker effects in Study 2. Although main effects of oxytocin on cognition and behaviour have been detected within the timeframe of Study 2 (Ellenbogen *et al.*, 2012; Berends *et al.*, 2019), the peak effects of oxytocin on regional cerebral blood flow were found from 39 to 51 min post-administration during arterial spin labelling magnetic resonance imaging (Paloyelis *et al.*, 2016), well before the time of testing in Study 2. Timing effects are further complicated by the fact that exogenous oxytocin appears to elicit changes in different brain regions depending on the time since drug administration (Martins *et al.*, 2020), which underscores the need for further research and a better understanding of the time course of oxytocin’s effects on cognition.

### General discussion

The current studies examined whether context-dependent (non-social *vs* social contact) and person-dependent factors (level of depressive symptoms) influence oxytocin’s effects on autobiographical memory recall. Of importance, the present studies are among the first to test the effects of oxytocin on memory recall during an experimental manipulation of social context in a controlled laboratory setting. The studies showed evidence of context-dependent effects of oxytocin on autobiographical memory, in that oxytocin administration increased ratings of vividness when administered in a social context, but not when the task was administered by computer across two studies. Context-dependent negative effects were also observed among vulnerable participants, in that oxytocin elicited the recall of negative memories in persons with elevated depressive symptoms during a non-social context, but not during the social context.

Overall, the present results build on previous research showing that oxytocin’s effects on social cognition and behaviour are sensitive to context (Bartz *et al.*, 2011; Cardoso *et al.*, 2016b). A number of studies report putative negative effects associated with the administration of intranasal oxytocin, including decreased trust and cooperation and increased defensive aggression, gloating and envy in situations that tend to be non-social or involve competition (Shamay-Tsoory *et al.*, 2009; Declerck *et al.*, 2010; De Dreu *et al.*, 2011, 2012; Fischer-Shofty *et al.*, 2013). Recent studies have shown that intranasal oxytocin, relative to placebo, increases aggressive responding and hostility during provocation tasks (Ne’eman *et al.*, 2016; Romney *et al.*, 2019). While some studies include explicit manipulations of context (Declerck *et al.*, 2010; De Dreu *et al.*, 2011, 2012), other studies refer to ‘contextual’ effects as a means of explaining consistency between study-specific contextual factors (i.e. exposure to aversive noise blasts as punishment) and oxytocin-induced behavioural changes (i.e. increased self-reported hostility; Ne’eman *et al.*, 2016; Romney *et al.*, 2019), with no actual manipulation of context. In contrast, the present study and a previous one (Cardoso *et al.*, 2016b) show context effects using a within-subject experimental manipulation of social context (being alone *vs* working with a research assistant).

The finding that oxytocin increases ratings of vividness of memory recall when administered in a social context is particularly noteworthy, in that it is consistent with a key conceptualisation of how oxytocin alters social behaviour by increasing the salience of social signals in the environment (Shamay-Tsoory and Abu-Akel, 2016). It is consistent with a large body of research showing that oxytocin improves emotion recognition in standard cognitive tasks (Ellenbogen, 2018; Schwaiger *et al.*, 2019). The finding also suggests that the salience effects observed for external social stimuli might also apply to internal processes such as the recall of personal memories, perhaps in the same way that oxytocin increased ratings of participants’ self-perception of their own personality (Bartz *et al.*, 2015). Taken together, oxytocin might promote social bonding in humans by increasing the salience of both social cues in the environment and internal self-perceptions such as social memories, but only when social contact is readily available.

In addition to context effects, the present findings further support the hypothesis that oxytocin has stronger effects in populations at risk for MDD and perhaps other mental health disorders associated with interpersonal dysfunction. There are a number of lines of evidence to support this view. Both low and elevated peripheral levels of plasma oxytocin have been reported in persons having MDD relative to non-depressed controls (Parker *et al.*, 2010; Massey *et al.*, 2016), perhaps reflecting chronic dysregulation of the endogenous system. Across different outcomes, we have shown that the administration of intranasal oxytocin alters selective attention and inhibition of emotional stimuli more strongly in persons reporting high, sub-clinical depressive symptoms than persons with low depressive symptoms (Ellenbogen *et al.*, 2012, 2013). Similar results have been reported in depressed participants, although only a few studies have been published to date (Pincus *et al.*, 2010; MacDonald *et al.*, 2013; Mah *et al.*, 2013). The acute administration of oxytocin in new mothers with MDD, relative to placebo, resulted in improved emotion recognition, higher ratings of the quality of relationship with their infants and a stronger protective response when confronted with a socially intrusive stranger (Mah *et al.*, 2013, 2015). Thus, the present findings further support the view that persons with MDD might show increased sensitivity to exogenous oxytocin, which may have important treatment implications. On one hand, the recall of more negative memories among persons with depressive symptoms following oxytocin administration provides further evidence of the potential deleterious effects of increasing oxytocin bioavailability in vulnerable populations in the absence of positive social contact. The present findings highlight the risk of using intranasal oxytocin as a potential adjunct to treatment in persons who are experiencing environmental adversity, such as social isolation or dysfunctional interpersonal relations. On the other hand, increased sensitivity to exogenous oxytocin among depressed patients might indicate the potential to improve therapeutic outcomes when the hormone is administered in a positive social context. Indeed, a pilot study found that oxytocin administered before psychotherapy sessions improved therapeutic outcomes in patients with MDD, relative to placebo administration, at post-treatment and 6-month follow-up, well after oxytocin use was discontinued (Ellenbogen *et al.*, 2018). Psychotherapy sessions could be considered a controlled and positive social context for persons with MDD, which might be ideal for using oxytocin therapeutically. Further study, however, is needed to explore the potential benefits and risks of using intranasal oxytocin as a possible adjunct to the treatment of MDD.

In a previous study, we found that intranasal oxytocin, relative to placebo, decreased the number of overgeneral memories recalled and increased the recall of positive social affiliation memories (Cardoso *et al.*, 2014b). However, irrespective of context, the finding of reduced overgeneral memories following oxytocin administration was not replicated across present studies. With respect to the finding of increased positive social affiliation memories, the present studies used a different coding scheme that did not assess this aspect of autobiographical memory. Given the current critiques of human research on oxytocin (Leng and Ludwig, 2016), it is important to highlight this discrepancy across the three studies assessing overgeneral episodic memories, which is a core cognitive deficit of MDD (Hermans *et al.*, 2008).

In addition to the possibility that the first result was a false positive, it is important to note that the participants of the Cardoso *et al.*, (2014b) study differed in important ways from the participants reported in the present manuscript. Their sample consisted of young men recruited to participate in a study assessing whether oxytocin dampens the stress response to physical exercise, and thus, one of the inclusion criteria was that participants be able to engage in 60 min of vigorous exercise (running) on a treadmill. The sample exercised, on average, for 6.9 h per week and thus represented a particularly healthy group of young men. In contrast, the two mixed-sex samples reported in the current manuscript had no such exercise requirement, and the Study 1 sample was recruited to have higher depressive symptoms. Although the effect of participants’ sex was not significant in the present studies, the inclusion of women could still have introduced noise into the data as there is growing evidence of sex differences in response to oxytocin (e.g. Xu *et al.*, 2020). Finally, participants in the Cardoso *et al.*, (2014b) study completed the autobiographical memory test 40 min after completing 60 min of exercise, which led to improved mood ratings post-exercise. It is possible that these factors influenced memory recall and/or sensitivity to oxytocin. Thus, the sample and procedures in their study and those in the current studies were quite different, which might account for the lack of consistency in oxytocin-induced changes in autobiographical memory across the three studies. Importantly, if our speculations are correct, the effects of oxytocin on overgeneral memory are highly sample- and/or context specific and, thus, unlikely to generalise to more typical populations.

#### Limitations and future directions.

A number of study limitations warrant consideration. A key limitation of the contextual manipulation is that participants disclosed autobiographical memories to a research assistant who is essentially a stranger, someone with whom the participant has only just met. Therefore, the procedures used in the studies may not reflect an ecologically valid test of normal social functioning in the natural environment, where people tend to seek social contact with persons they know or desire to know better. Study 2, however, provides a basic manipulation of social context while minimising a number of potentially confounding extraneous factors (i.e. relationship quality, length and type) that would complicate a more ecologically valid paradigm, such as having intimate partners or friends elicit autobiographical memories in study participants.

Another limitation of the studies is that the pathway by which intranasal oxytocin influences social cognition remains unknown, as this methodology increases bioavailability not only in the brain, but also in periphery. There have been questions raised about the effectiveness of the intranasal methodology in directly reaching brain targets (Leng and Ludwig, 2016). However, there is increasing evidence of nose-to-brain transport of oxytocin and similar molecules into the brain via the intranasal route (Quintana *et al.*, 2018; Martins *et al.*, 2020). Still, more research is needed to substantiate the precise pathway, appropriate dosage and optimal timing to maximise oxytocin’s effects in the brain.

Finally, the results of the current studies should be interpreted cautiously. Despite the positive finding for vividness ratings of recalled memories, no other drug-related changes on memory characteristics were found, which raises the possibility of a Type I error. The drug by depression interaction fell short of conventional levels of statistical significance for one of the two studies. However, the relationship was consistent across two independent samples, and the studies reported similar effect sizes. Still, further replication of both reported findings in larger samples will be required to substantiate these results.

## Conclusion

The current studies show that oxytocin promotes vivid memory recall and further support the view that oxytocin’s effects on social cognition are dependent on contextual factors. There are important clinical implications to consider. The present studies demonstrate the negative impact of administering intranasal oxytocin in persons at risk for MDD when no social contact is readily available. Given that clinical trials of intranasal oxytocin are underway in different clinical populations, the present findings suggest that the availability of a positive social outlet is necessary to elicit the prosocial effects of exogenous oxytocin. Using oxytocin therapeutically in socially isolated depressed populations, for example, may be contraindicated. However, if the administration occurred in a controlled positive social setting, such as a psychotherapy session, perhaps the hormone could help improve the treatment of MDD. Further research is required to test this speculative hypothesis.
